# Sample size recalculation based on the prevalence in a randomized test-treatment study

**DOI:** 10.1186/s12874-022-01678-7

**Published:** 2022-07-25

**Authors:** Amra Hot, Norbert Benda, Patrick M. Bossuyt, Oke Gerke, Werner Vach, Antonia Zapf

**Affiliations:** 1 Institute of Medical Biometry and Epidemiology, University Medical Center Hamburg-Eppendorf, Christoph-Probst Weg 1, 20246 Hamburg, Germany; 2 Federal Institute for Drugs and Medical Devices (BfArM), Kurt-Georg-Kiesinger-Allee 3, 53175 Bonn, Germany; 3 Department of Epidemiology and Data Science, Amsterdam University Medical Centers, Meibergdreef 15, Amsterdam, 1105 AZ The Netherlands; 4 Department of Nuclear Medicine, Odense University Hospital, J.B. Winsløws Vej 4, 5000 Odense C, Denmark; 5 Department of Clinical Research, University of Southern Denmark, Winsløwparken 19, 5000 Odense C, Denmark; 6 Basel Academy for Quality and Research in Medicine, Steinenring 6, 4051 Basel, Switzerland; 7 Department of Environmental Science, University of Basel, Spalenring 145, 4055 Basel, Switzerland

**Keywords:** Adaptive design, Sample size recalculation, Sensitivity, Specificity, Prevalence

## Abstract

**Background:**

Randomized test-treatment studies aim to evaluate the clinical utility of diagnostic tests by providing evidence on their impact on patient health. However, the sample size calculation is affected by several factors involved in the test-treatment pathway, including the prevalence of the disease. Sample size planning is exposed to strong uncertainties in terms of the necessary assumptions, which have to be compensated for accordingly by adjusting prospectively determined study parameters during the course of the study.

**Method:**

An adaptive design with a blinded sample size recalculation in a randomized test-treatment study based on the prevalence is proposed and evaluated by a simulation study. The results of the adaptive design are compared to those of the fixed design.

**Results:**

The adaptive design achieves the desired theoretical power, under the assumption that all other nuisance parameters have been specified correctly, while wrong assumptions regarding the prevalence may lead to an over- or underpowered study in the fixed design. The empirical type I error rate is sufficiently controlled in the adaptive design as well as in the fixed design.

**Conclusion:**

The consideration of a blinded recalculation of the sample size already during the planning of the study may be advisable in order to increase the possibility of success as well as an enhanced process of the study. However, the application of the method is subject to a number of limitations associated with the study design in terms of feasibility, sample sizes needed to be achieved, and fulfillment of necessary prerequisites.

**Supplementary Information:**

The online version contains supplementary material available at 10.1186/s12874-022-01678-7.

## Background

The patient’s health should be the primary consideration when evaluating diagnostic tests [1, 2]. Once a diagnostic test is approved, randomized controlled trials are needed in order to evaluate the clinical effectiveness of a diagnostic test [1, 3]. These trials are stated as randomized test-treatment trials where patients are randomized to diagnostic test procedures and subsequently receiving treatments. Finally, patient-relevant outcomes are evaluated, leading to evidence of potential patient benefit, such as reduction in patient mortality or morbidity or improvement in health-related quality of life. In this setting, “test-treatment” pathways are assessed rather than interventions alone [4–6].

**Fig. 1:**
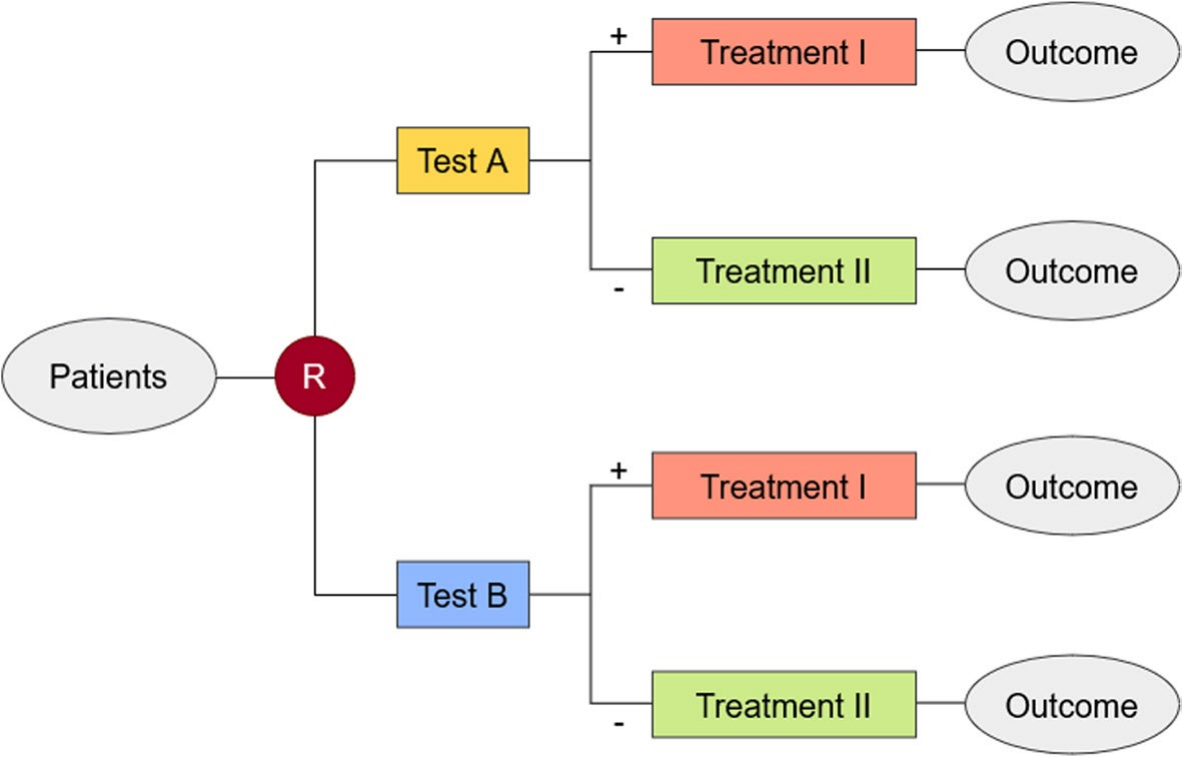
A schematic representation of a classical randomized test-treatment study [4, 5]

In principle, patient outcomes are affected by a linkage between a test result and subsequent treatment choices. One commonly referred design is the classical two-arm design, in which patients are randomized to two test-treatment arms [4, 7]. In one arm, an experimental test *A* is applied, in the other a comparator test *B*. Test results are communicated and associated with subsequent management decisions: for example, test positives are assigned to treatment *I* and test negatives to treatment *II*. Afterwards, the patient outcome is evaluated in each subgroup (see Fig. 1). Further designs are introduced in the literature, but will not be discussed here [4, 7]. So far, only a few randomized controlled trials (RCTs) that evaluate diagnostic strategies on patient outcome are published [4, 8]. One example is a randomized trial to evaluate and compare the safety of two management strategies based on repeated Ultrasonography and D-Dimer testing in patients with suspected deep vein thrombosis [9]. Another trial investigated two different diagnostic approaches for the management of outpatients with dysphagia who have a high risk for developing aspiration pneumonia [10].

Randomized test-treatment trials may involve a few challenges in terms of study planning and practical feasibility. One challenging aspect is the calculation of the required sample size which is an essential step in the planning of any clinical trial. A fixed clinical trial is designed with a targeted sample size that is specified at the planning stage and the study is conducted according to its pre-specified study protocol without prospectively planned adaptations. After data collection is completed, data are analyzed following a predefined statistical analysis plan. In the design phase of the randomized test-treatment study, the total number of participants needed to detect a clinically relevant test-treatment difference with sufficient power depends on assumptions about the expected treatment effect, in addition to the type I error rate and power, as well as parameters such as disease prevalence and diagnostic test accuracy [3]. However, reliable estimates of these variables are often weak guesses based on barely reliable empirical information. Therefore, there is a high risk of not reaching the targeted power in a fixed sample size design. In this sense, it may be worth considering adjustments during the study regarding wrong assumptions to increase the chance of a successful and efficient study, thus requiring the implementation of an adaptive trial design in order to make the clinical trial more flexible [11, 12]. “An adaptive design is defined as a clinical study design that allows for prospectively planned modifications based on accumulating study data without undermining the study’s integrity and validity” [13]. They can be applied across all phases of clinical research, from dose escalation to confirmatory trials. One possible adaptation is sample size recalculation (SSR) in a blinded manner based on accumulating interim data from an internal pilot study. Blinding the study until its finalization means that no differences between the study arms can be observed until then, nor can null hypotheses be tested [14]. Some methods for interim sample size adjustment require unblinding of the treatment assignment [15], while other methods do not [16, 17].

In general, adaptive designs in therapeutic trials, including blinded SSR, are well accepted by regulators [13]. However, adaptive study designs are much less common in diagnostic trials than in intervention trials [18]. Research for blinded sample size recalculation in the context of intervention trials does already exist [19–23], but is barely existent in randomized test-treatment studies [4]. Stark and Zapf [17] proposed an optimal sample size calculation procedure and dealt with blinded sample size recalculation in the context of single-arm confirmatory diagnostic accuracy studies. To our knowledge, the present paper is the first to describe an approach using interim data from a randomized test-treatment study which generally does not result in the trial being unblinded before its completion and therefore results in no or negligible inflation of the probability of falsely rejecting the null hypothesis.

In the scope of this paper, an adaptive design for recalculating the sample size is presented by addressing the internal pilot study approach proposed by Wittes and Brittain [24], which can as well be applied to randomized test treatment trials. Basically, the data from the first part of the patients included in the study are used to estimate nuisance parameters that are essential for this study design. Depending on the results in the internal pilot study, the study may be adapted before the study is continued. The final analysis includes all recruited patients, including those from the internal pilot study. The decision of a suitable nuisance parameter depends on the specific study design as well as on the primary endpoint chosen in the study. In trials with a binary primary endpoint, the overall response rate is usually used to estimate the sample size in a blinded manner. Assuming a continuous endpoint, the pooled variance may be used. The expected outcome in randomized test-treatment trials is derived from a set of parameters that can be considered as nuisance parameters [4]. Here, the disease prevalence is chosen as a nuisance parameter which implies the application of a reference standard to estimate the proportion of diseased individuals in the interim analysis. The adaptive design is hence defined by using this prevalence estimate for a recalculation of the sample size.

The overall objective of this work is to investigate by means of a simulation study whether

- the adaptive design reaches its goals, i.e. the actual power equals the pre-specified power and the type I error rate is controlled
- the recalculated sample sizes are feasible
- the gap between nominal and actual power in the fixed design is substantial and hence justifies the additional efforts implied by using an adaptive design.

The paper is structured the following way. The methods section comprises (1) a theoretical basis for the sample size calculation in a classical randomized test-treatment study; (2) an example study; (3) the proposed procedure of a single, blinded sample size recalculation based on the prevalence applied to the example; and (4) the practical implementation within a simulation study. Afterwards, the results of the simulation study are presented according to the above stated objectives. Finally, we close with a discussion and brief conclusion.

## Methods

### Design considerations

In the following, we consider a classical randomized test-treatment trial evaluating a binary patient outcome, e.g. mortality, and refer to the notation in Hot et al. [4]. In general, test-treatment strategies are compared where the particular test is linked to the treatments by the test results. Firstly, let *D* ∈ {+, −} be the true disease status of the individuals included in the trial, where *D* = + denotes those with the target condition (i.e. the truly diseased) and *D* = − those without. Hence, *π* = *P*(*D* = +) refers to the proportion with the target condition in the study. If the target condition implies the presence of a disease, then *π* indicates the disease prevalence of the population. Ideally, the determination of the patient’s target condition or disease state is carried out using a reference standard, a diagnostic procedure that is applied to the study population in addition to the investigated testing procedures. In case no reference standard can be obtained, alternative methods have to be considered in order to approach the disease prevalence. In the first phase of the trial, all individuals of interest are equally randomized to two (diagnostic) tests *A* and *B* with binary response, where test *A* is an experimental test and B a comparator test (Fig. 1). Let *T* denote the test applied to a patient and *R*_*Τ*_ ∈ {+, −} the result of the corresponding test *Τ* ∈ {*A*, *B*}. After receiving the test results, the intervention (management strategy) *M* ∈ {*I*, *II*} is given to the patient based on the respective test result, i.e. *M* = *m*(*R*_*Τ*_ ) with *m*(+) = *I* and *m*(−) = *II*. Management strategy *I* may be a more invasive treatment or therapeutic approach which should work better for truly diseased patients, and management *II* may represent a standard of care which should work better in truly non-diseased patients. Finally, after receiving a treatment strategy the patient relevant binary response variable *Y* is measured. The clinically relevant hypotheses of interest can be expressed in terms of the difference of the event response rates *Δ* = *θ*_*A*_ − *θ*_*B*_ following in the next lines:

$${H}_{0}:\varDelta =0\ vs.{H}_{1}:\varDelta \ne 0$$

with *θ*_*t*_ ≔ *E*(*Y*| *Τ* = *t*) denoting the expected outcome/event response rate in each test-treatment arm based on test *T* = *A*, *B*. Subsequently, the expected outcomes for test *T* = *t* can be expressed as

1 $${\theta}_{t}=\sum\limits_{r_{t}\in \left\{+,-\right\},d\in \left\{+,-\right\}}{\mu}_{m\left({r}_{t}\right){r}_{t}d}^{t}P\left({R}_{t}={r}_{t}|D=d\right)P\left(D=d\right)$$

with $\mu_{m\left({r}_{t}\right)r_{t}{d}}^{t}=E\left(Y|M=m,{R}_{t}={r}_{t},D=d\right)$ denoting the expected outcome in the respective subgroup of patients [4], *P*(*R*_*t*_ = *r*_*t*_| *D* = *d*) referring to the sensitivity and specificity of the two tests, i.e. the proportion of truly diseased and non-diseased patients, respectively, who are correctly identified and *P*(*D* = *d*) presenting the proportion of patients with and without the target condition. The following applies to all further considerations: the sensitivity and specificity of a test *t* = *A*, *B* are denoted as *Se*_*t*_ ≔ *P*(*R*_*t*_ = +| *D* = +) and *Sp*_*t*_ ≔ *P*(*R*_*t*_ = −| *D* = −), respectively.

The sample size for binomial trials depends on the response rates assumed under the null and alternative hypotheses for each arm, the type I error rate, and the power. The sample size *N* = *N*_*A*_ + *N*_*B*_ needed for this trial design can be calculated for the balanced design by inserting *θ*_*A*_ and *θ*_*B*_ in the following formula

2 $${N}_{A}={N}_{B}={\left[\sqrt{2\overline{\theta}\left(1-\overline{\theta}\right)}{z}_{1-\, {}^{\alpha }\!\left/ {}_{2}\right.}+\sqrt{\theta_{A}\left(1-{\theta}_{A}\right)+{\theta}_{B}\left(1-{\theta}_{B}\right){z}_{1-\beta }}\right]}^{2}/{\varDelta}^{2}.$$

Here, the term

3 $$\overline{\theta}=\frac{\left({\theta}_{A}+{\theta}_{B}\right)}{2}$$

denotes the overall response rate [25].

Because this article is a continuation of previous work [4], we have again formulated the methods for a binary endpoint. However, they can be easily applied to continuous endpoints. In this case, additional assumptions concerning the variation of *Y* are needed, which is usually a nuisance parameter in the context of blinded sample size recalculation [21, 23, 26, 27]. The R-code for the example is provided in the electronic supplement material.

### Example study

In order to illustrate the sample size calculation in a randomized test-treatment study, a multicenter RCT for the evaluation of point-of-care Xpert MTB/RIF testing for tuberculosis compared with smear microscopy is presented [28]. In total, 1502 adults with symptoms suggestive of active tuberculosis from primary-care health-care facilities in Africa were randomized to nurse-performed Xpert MTB/RIF at the clinic or sputum smear microscopy. At recruitment, at least two spot expectorated sputa were obtained sequentially from each patient. One specimen, selected randomly, was used for smear microscopy or Xpert MTB/RIF. The other specimen underwent culture. Culture for M tuberculosis was used as the reference procedure. If a positive smear microscopy or Xpert MTB/RIF result was obtained, the patient was referred to the tuberculosis treatment office at the same clinic. Patients who were smear-negative or Xpert MTB/RIF-negative were referred (with their chest radiographs) for routine clinical review. The primary outcome was tuberculosis-related morbidity, measured with the TBscore and Karnofsky Performance Score (KPS). A higher TBscore and a lower KPS score indicate more morbidity. In the sample size planning, it was calculated that 63 culture-positive patients in each group are needed to detect a statistical difference of 1 point in TBscore and a difference of 10 points in KPS between the two study arms with a power of 80% and a two-sided type I error rate of 5%. Assuming a dropout rate of 30% and an overall study tuberculosis prevalence of 15%, in total 550 patients were to be recruited in each arm.

### A hypothetical follow-up study

In the following, we consider a hypothetical follow up study with a sample size calculation partially informed by the results of by Theron et al. [28]: the sensitivity and specificity for Xpert MTB/RIF are assumed to be 88 and 98%, i.e. *Se*_*Xpert*_ = *P*(*R*_*Xpert*_ = +| *D* = +) = 0.88 and *Sp*_*Xpert*_ = *P*(*R*_*Xpert*_ = −| *D* = −) = 0.98, and for smear microscopy 50 and 96.5%, i.e. *Se*_*smear*_ = *P*(*R*_*smear*_ = +| *D* = +) = 0.50 and *Sp*_*smear*_ = *P*(*R*_*smear*_ = −| *D* = −) = 0.965.

In the following, all considerations refer to a continuous outcome, i.e. TBscore. We use formula (1) and compare in the primary hypothesis the mean responses based on the test-treatment strategy *A* and *B*. Accordingly to the binomial case, the difference in mean responses is *Δ* = *θ*_*A*_ − *θ*_*B*_, where the hypothesis of interest is written as *H*_0_ : *Δ* = 0 *vs*. *H*_1_ : *Δ* ≠ 0. The formula for calculating the sample size of a continuous outcome comparing two independent means is used according to Chow et al. [25].

For both arms, we assume that, if a diagnosis is correctly made (true positive), then TBscore is 2 points. If patients are correctly classified to be tuberculosis free (true negative), then the TBscore is assumed to be 1 point. Among patients who are falsely diagnosed (false positive), the TB score indicates 4 points. For those patients in whom a diagnosis remains undetected (false negative), the TBscore is estimated to be 5 points. The overall rate of tuberculosis prevalence is assumed to be 15% in the study population, i.e. i.e. *P*(*D* = +) = 0.15. To test the null hypothesis of no difference in outcome (TBscore) between the groups, we set the type I error at 5% and the power at 80%. Under the above assumptions and assuming a common standard deviation of 2 points, the Xpert MTB/RIF testing group is expected to have a reduction in TBscore by 0.2 points compared to the smear microscopy group. A two-sided t-test for comparing two independent means is used to calculate the sample size. In total, *N* = 3,142 patients are needed to show a difference in the TBscore between the two groups.

### Blinded sample size recalculation

At the planning stage of a randomized test-treatment trial, sample size calculation is based on essential information that may not be available or highly uncertain. In case of unreliable assumptions made at the initial stage of a clinical trial, it may be wise to check the validity of those assumptions using interim data from the study. The basic idea of a blinded sample size recalculation is to conduct one interim analysis without unblinding the test-treatment assignment to provide an estimate of a nuisance parameter. In this context, the prevalence *π* = *P*(*D* = +) constitutes the nuisance parameter which is used to adjust the sample size in order to preserve the power without affecting the type I error rate and prevent unblinding the study. For the estimation of the prevalence, the assessment of the reference standard for the study population is required.

The following steps are considered [24]:

- In the first step, using the sample size formula in (2), the initial sample size *N*_0_ is calculated based on assumptions regarding the sensitivity and specificity of the two diagnostic tests A and B, i.e. *Se*_*A*_, *Se*_*B*_, and *Sp*_*A*_, *Sp*_*B*_, respectively, as well as assumptions regarding the expected outcome ${\mu}_{m\left({r}_{t}\right){r}_{t}d}^t$ with *r*_*t*_, *d* ∈ {+, −}, *m*(*R*_*t*_) ∈ {*I*, *II*} and *t* ∈ {*A*, *B*}. Additionally, an assumption regarding the prevalence, *π*_*assumed*_ on the nuisance parameter *π* is needed.
- In the second step, patients are recruited until a predetermined fraction (*f*) of the initial sample size *N*_0_, denoted by *N*_1_ = *N*_0_ ∙ *f*, is obtained. At the interim stage of the trial, the nuisance parameter is estimated. Suppose that that n patients out of *N*_1_ observations are diagnosed as having the target condition defined by the reference standard, so that
  $$\hat{\pi}=n/{N}_{1}$$

estimates the prevalence π. The estimator represents the maximum likelihood estimator of a binomial proportion [29].

Substituting $\hat{\pi}$ for *π* in (2) provides the re-calculated sample size *N*^∗^ that the current data suggest should have been specified for the trial. If the re-calculated sample size *N*^∗^ is larger than the already recruited sample size *N*_1_, further patients will be recruited until the adjusted sample size will be reached. Otherwise, no further recruitment beyond *N*_1_ is necessary. Finally, the study is analyzed based on the unadjusted type I error level due to the blinded character of the recalculation procedure.

#### Application to the hypothetical follow-up study

After 50% of all originally intended 3142 participants have been recruited, a sample size recalculation based on the estimated prevalence resulting from the reference standard information, i.e. the culture positivity for M tuberculosis complex, is performed. Let us assume that we observe a prevalence 25%. Based on this, the recalculated sample size is equal to 2012, i.e. distinctly smaller than the intended sample based on an assumed prevalence of 15%. This reflects that with a higher prevalence, the advantage of Xpert MTB/RIF (the much higher sensitivity) becomes more visible in the study results. Therefore, based on these assumptions, a remaining number of 441 patients need to be included in addition to the number already recruited in order to reach the trial objective.

### Implementation of the simulation study

A simulation study was conducted to assess the method of sample size recalculation in the context of a randomized test-treatment study by investigating different scenarios. For each scenario, we consider the bias, the type I error and the actual power of the adaptive design in order to examine, whether the adaptive design achieves the pre-specified power and do not inflate the type I error rate. In particular, the empirical type I error rate is calculated as proportion of *p*-values from testing the null hypothesis of no difference on each simulated sample that are less than the 5% significance level, when the null hypothesis is true. The power is determined as the proportion of simulation samples in which the null hypothesis of no effect is rejected at the two-sided 5% significance level, when the null hypothesis is false. In the Supplementary Material (see Additional file 1), we report the calculated bias of the estimated prevalence in the interim analysis as percentage of the true value, i.e. $\left(\frac{\overline{\pi}-\pi }{\pi}\right)\cdot 100\%$ [30] to verify an unbiased estimation of the prevalence in the interim analysis. In order to examine the feasibility of the sample sizes, we show the distribution of the true necessary, initial, and recalculated sample sizes across all scenarios. In addition, for a selected scenario, we report how much the recalculation sample sizes deviate from the true necessary sample size as well as from the initial sample size across all simulation runs. With respect to illustrate the gap between nominal and actual power when not using an adaptive design, we report also the power of the fixed design. We present the distribution of these quantities across all scenarios using boxplots stratified by selected parameters.

In total, 1620 scenarios were simulated, i.e. two sets of the true prevalence, five sets of the assumed prevalence, three sets each from the sensitivity and specificity of test A as well as test B under the null and alternative hypothesis, respectively, and two sets of the expected outcome in diseased patients receiving treatment I. The variations of the parameters are given in Table 1 in the Supplementary Material (see Additional file 1). Per scenario, 10,000 replications were performed. In order to limit the complexity of the simulation study, we assumed that the expected outcomes${\mu}_{m\left({r}_t\right){r}_td}^t$ with *r*_*t*_, *d* ∈ {+, −}, *m*(*R*_*t*_) ∈ {*I*, *II*} and *t* ∈ {*A*, *B*} in the test-treatment arms were independent of the applied tests, i.e. the effect of a correct diagnosis (and consequently correct treatment) as well as a false diagnosis was assumed to be the same in both diagnostic groups, respectively. Thus, we only considered the expected outcome of treatment I and II in dependence of the true disease state of the patient, i.e. *μ*_*md*_ with *d* ∈ {+, −}, *m* ∈ {*I*, *II*}.

Provided that decreasing effects are favorable (e.g. lower mortality), we assumed that treatment I induced a positive/curative effect in the diseased population, so that the expected rate in this group should be considered quite low, i.e. μ_I+_ = 0.05 or 0.1. Similarly, if the non-diseased individuals received the treatment strategy that was optimal for them, i.e., treatment II, a low event rate could be expected, i.e. *μ*_*II*−_ = 0.05. Patients who tested falsely positive unnecessarily underwent an invasive treatment, leading to potential complications or side effects, resulting in a high event rate, i.e. *μ*_*I*−_ = 0.2. Patients who tested falsely negative and did actually have the disease did not receive the treatment they needed, which may lead to disease-related complications and consequences, suggesting a high event rate, i.e. *μ*_*II*+_ = 0.25. In addition, it was defined in the simulation that under the alternative hypothesis, apart from prevalence, the true values for the diagnostic accuracy parameters as well as the true expected outcomes were assumed in the sample size recalculation.

Statistical significance was assessed using the Wald-test for comparing two independent binomial proportions [31, 32]. The simulation was performed using R (Version 4.0.1) [33]. The simulation program as well as a list of R packages used are given in the Supplementary Material (see Additional file 2).

### An alternative approach to estimate the prevalence in a blinded manner

In case no reference standard is available for all study participants or is not available at all and, additionally, if we make reliable assumptions about the diagnostic accuracy of the tests applied, the nuisance parameter can be estimated by calculating the overall positive rate in the interim analysis to then resolve the following equation according to the nuisance parameter *π* for *d* ∈ {+, −}:

4 $${\displaystyle \begin{array}{c}{P}^{+}:= \sum\limits_{t=A,B}P\left({R}_{t}=+\right|\ S{e}_{t},S{p}_{t},D=d\Big)\ \\ {}=\pi \cdot S{e}_{A}+\left(1-\pi \right)\cdot \left(1-S{p}_{A}\right)+\pi \cdot S{e}_{B}+\left(1-\pi \right)\cdot \left(1-S{p}_{B}\right)\end{array}}$$

where *P*^+^ is the probability of obtaining a positive test result in the study based on the diagnostic accuracy of test *A* and *B* and disease prevalence. It follows:

5 $$\pi =\frac{P^{+}+S{p}_A+S{p}_B-2}{\left(S{e}_A+S{p}_A-1\right)+\left(S{e}_B+S{p}_B-1\right)}$$

with *Se*_*A*_ + *Se*_*B*_ + *Sp*_*A*_ + *Sp*_*B*_ − 2 ≠ 0 and 0 ≤ *P*^+^ ≤ *Se*_*A*_ + *Se*_*B*_.

However, this approach is not investigated in this paper.

## Results

In this section, we present the results of the simulation study to illustrate whether the adaptive design reaches the pre-specified power of 80% and controls the type I error rate. With respect to illustrate the gap between nominal and actual power when not using an adaptive design, we also report the power and type I error rate of the fixed design.

Simulation results presenting the distribution of the achieved power and type I error rate are visualized by means of boxplots stratified by different scenarios, in particular depending on the difference between the assumed and true prevalence as well as the difference in diagnostic accuracy of the two tests applied. In addition, a distinction is made between an expected treatment effect of treatment I in the diseased population by setting *μ*_*I*+_ = 0.05 and 0.1. Ideally, power and the type I error should be maintained over all simulated scenarios. In order to obtain an overview of the feasibility of the design, i.e. whether the calculated sample sizes are realistic, the distribution of sample sizes for all simulated scenarios, stratified according to the initial, adjusted and true necessary sample size calculation, is also shown graphically. In addition, the bias in estimating the prevalence is considered.

**Fig. 2:**
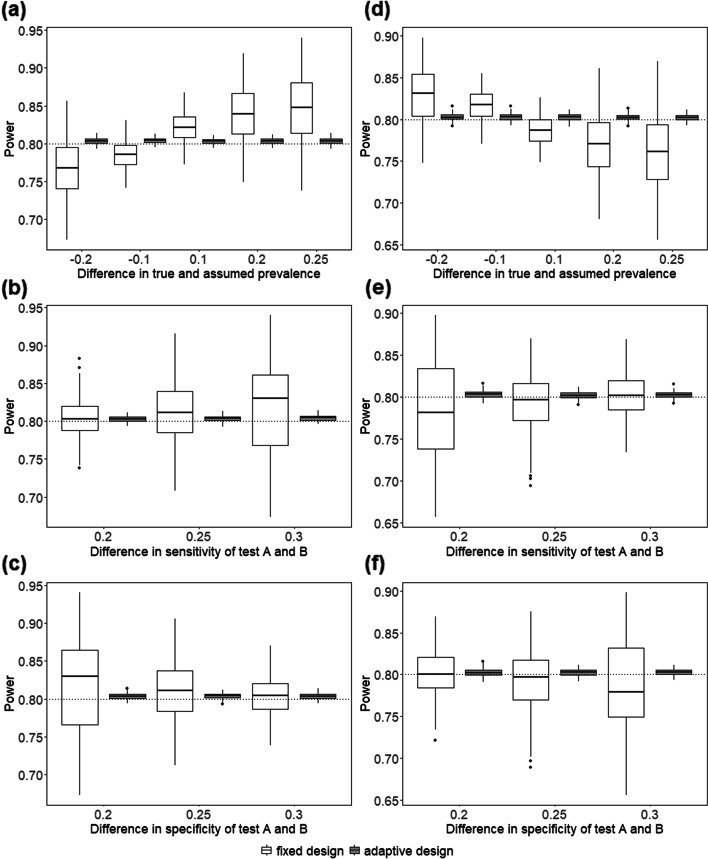
Results for the power for the 1620 scenarios stratified by the difference in prevalence, the difference in sensitivity, and the difference in specificity. The power of the fixed design was compared to the adaptive design containing a re-estimation of the prevalence assuming *μ*_*I*+_ = 0.05 (**a**-**c**) and *μ*_*I*+_ = 0.1 (**d**-**f**). The black dotted line mark the theoretical power of 80%. The black bold line in the box marks the median value

In Fig. 2, the results of the simulation study reveal that the desired theoretical power of 80% was reached in the adaptive design independently of the simulated scenarios. The median power in the adaptive design across all scenarios is 0.8032, and the power is generally located tightly around the nominal level. The re-estimation of the prevalence diminished the effect of an initially wrongly specified prevalence.

In the fixed design, the calculated power varies in relation to an over- and underestimation of the true prevalence, the magnitude of the difference between sensitivity and specificity of test A and B as well as the choice of *μ*_*I*+_. In case of *μ*_*I*+_ = 0.05, an underestimation of the true prevalence leads to an underestimation of the power in the fixed sample size design for more than 50% of the scenarios, thus requiring subsequent recruitment of further patients than originally planned (Fig. 2a). Analogously, an overestimation of the true prevalence resulted in the recruitment of fewer patients than originally planned in the fixed design. The larger the difference between the true and assumed prevalence, the greater the dispersion of the power around the median value in the fixed design. Similarly, the results of the power were affected by the assumed differences in sensitivity and specificity between Test *A* and Test *B*. The larger the difference in sensitivity between the two tests, the greater the variability of the power and higher the median value in the fixed design (Fig. 2b). Both an increasing difference in sensitivity between the two tests and an underestimation of prevalence lead to a more pronounced overestimation of the power in the fixed design. When considering the specificity of the two tests, the dispersion of the calculated power in the fixed design as well as the median value across all scenarios decreases as the difference between test *A* and *B* increases (Fig. 2f). By choosing *μ*_*I*+_= 0.1 instead of 0.05, the patterns are reversed (Fig. 2d-f).

Supplementary Fig. 4 gives a more detailed look at the power for one specific scenario.

Supplemental Fig. 2 illustrates the results of empirical type I error rates for the adaptive and fixed designs for all 1620 simulated scenarios. It reveals that in nearly all scenarios the empirical type I error rate was controlled in the adaptive design as well as in the fixed design. Only for about 7% of the scenarios, the observed type I error rate was outside of the 95% prediction intervals, which may be due to chance. In addition, the distribution of the observed type I error rates did not depend on the sets of scenarios and thus was not affected by an overestimation or underestimation of the true prevalence nor by the magnitude of the difference between sensitivity and specificity of test *A* and *B* as well as the choice of ${{\mathsf{\mu}}}_{{\mathsf{\it I}}+}$.

The sample size results shown in Supplementary Fig. 3 demonstrate that, regardless of variation in prevalence, sensitivity, or specificity, the adjusted (recalculated) sample sizes tended to approach the true necessary sample size. The distribution of the initial sample size varies in dependence of the different parameters. Overall, sample sizes varied between 900 and 4000 individuals.

In order to assess the magnitude of the gain from recalculating the sample size in the interim analysis, we look at how much the recalculated sample size deviates from the true necessary sample size as well as from the initial sample size across all simulation runs, when *Se*_*A*_ = 0.95, *Sp*_*A*_ = 0.9, *Se*_*B*_ = 0.7, *Sp*_*B*_ = 0.75 and *π* = 0.4, *π*_*assumed*_ = 0.2. On average, the recalculated sample size differs from the true necessary sample size by a factor of 0.99 (SD = 0.0065) and from the initial sample size by a factor of 1.13 (SD = 0.0066) (Supplemental Fig. 5).

Supplemental Fig. 1 contains the results of the relative bias for the adaptive design of the scenarios with the same parameters as described above in the context of the Type I error rate and power. The prevalence of the re-estimated prevalence is estimated with almost no bias, and the median bias is approximately the same across all simulated scenarios.

## Discussion and conclusion

This paper shows a first approach of how an adaptive design, i.e. blinded sample size recalculation, can be integrated within a randomized test-treatment trial. Consequently, two important aspects are combined: considerations concerning the sample size calculation in randomized test-treatment studies and the application of an adaptive design. Randomized test-treatment studies involve the use of a twofold intervention, i.e., first the application of diagnostic tests and then the evaluation of subsequent treatments. A crucial element in the initial planning phase of a study is the sample size planning. In comparison to standard RCTs, parameters such as prevalence and diagnostic accuracy of the respective tests are required in addition to assumptions regarding the expected patient outcomes in the study arms. Due to lack of empirical information regarding these parameters, there is always a huge uncertainty at the planning stage of the trial. An adaptive design with a recalculation of the sample size as investigated here provides a first minor step how to overcome a part of lack of information regarding these parameters and may avoid unnecessarily recruiting too many patients at the end of the study – or too few.

Our first stated objective (a) in Background section is satisfied, i.e. the empirical power of the adaptive design equals the desired theoretical one of 80%. The type I error rate is not inflated and the prevalence is re-estimated without any bias. This recalculation procedure corrects a wrongly assumed prevalence and its consequences on the initial sample size. However, the results of our simulation studies demonstrate that an incorrectly specified prevalence can lead to under- or overpowered studies. The magnitude of this effect may be even larger in studies with varying treatment effects only in the diseased or non-diseased subpopulation, as this implies a higher impact of a miss-specified prevalence. A different set of scenarios, especially different treatment effects, may imply that the calculated sample sizes are unrealistically large, thus the implementation of such studies is not feasible and our second stated objective (b) is not always fulfilled.

The performance gain resulting from the implementation of an adaptive design is guided by the difference between the true necessary sample size and the recalculated as well as original sample size. When deciding whether such a gain should be considered clinically relevant and have an impact in practice, it requires to take into account also the impact for the patients, both in terms of outcomes and exposure to the interventions. This should be considered alongside the cost and effort involved in an adaptive design.

Further critical aspects arise that may complicate the use of this method in practice and need to be considered first: recalculation of sample size based on the prevalence as presented here involves the existence of a reference standard, i.e. a third diagnostic procedure has to be measured additionally. This requires the rapid measurement and unblinding of the reference standard test results, which may lead to an ethical problem in some cases. In particular, it becomes difficult to justify, in the knowledge of the reference standard, withholding the right treatment from patients who have tested falsely negative.

In order to weigh this problem, one must distinguish how severe the primary endpoint of interest is and whether it occurs in short-term or long-term.

In case of a substantial time gap between the administration of treatment based on the results of test *A* and *B* and the evaluation of the reference standard, the ethical problem mentioned could be circumvented. Here, one treatment strategy could correspond to “waiting for the reference result” and the other treatment is based on test A or B aiming to compare immediate treatment versus delayed treatment decision.

In practice, however, it is often difficult to find studies in which a reference standard is additionally obtained to evaluate the tests under examination. If this is the case, i.e. no reference standard can be additionally obtained, the interim estimation of the prevalence may be based on the proportion of all patients tested positive, provided that the test results are unblinded. Here we can either assume that the sensitivity and specificity of the tests under consideration are high allowing to approximate the prevalence by the proportion of test positives, or we can assume that sensitivity and specificity are known exactly allowing to use the approach outlined in An alternative approach to estimate the prevalence in a blinded manner section. The performance of these alternatives has still to be investigated in simulation studies.

Another crucial aspect of the classical two-arm design in general is that only those patients with discordant test results will actually affect the magnitude of the effect size [34]. In this context, it is worth considering already during the study planning stage whether it is practically possible and ethically reasonable to choose an alternative design in which both tests are applied in all patients and subsequently only patients with discordant test results are randomized. This study design can only be reasonably applied in a setting where both tests can be performed simultaneously, or where the application of one test would not influence the other. Elsewhere, we have elaborated the advantages and disadvantages as well as sample size planning of the classical design and the so called discordance design [4]. Based on the research question, one design can be preferred over the other. The presented adaptive design here can also be applied on the discordance design with additional assumptions regarding the discordance fraction.

In practice, it is difficult to find studies that provide exactly the information needed for sample size calculation. The prevalence of a disease can be obtained from empirical data as well as the diagnostic accuracy of a test from diagnostic accuracy studies. It is more difficult to obtain information on the treatment effects in the different subgroups of diseased and non-diseased individuals, as corresponding RCTs are lacking or even ethically not justifiable. In this work, we made already the simplifying assumption of no interaction between test and treatment.

In the simulation study, a simple comparison of rates was assumed in the primary analysis. In practice, the primary analysis may be enhanced by further prognostic factors that influence the overall outcome and/or have informed the randomization process, resulting into the use of some type of regression model.

In general, we have to acknowledge that the simulation study presented in this paper as well as the proposed study concept of an adaptive design is based on several simplifying assumptions and thus entails limitations. First, for feasibility reasons, we did not assume any interaction between treatment and test results. Because the simulation study and the associated results, which are already very complex, would be even more extensive, complex and difficult to present. Secondly, the procedure proposed here assumes that all parameters except prevalence are exactly known. This is a limitation of a blinded re-estimation of the sample size in favour of not risking inflation of the type I error and still being allowed to use additional useful information in the course of the study. However, possible and realistic deviations of the remaining parameters from the true value needs to be accepted. On the other hand, to perform an unblinded re-estimation of the sample size based on additional parameters estimated in the study the type I error would have to be adjusted for multiplicity. In practice, the benefits and harms of the different designs must be weighed against each other.

Further empirical and theoretical research is needed to show that our conclusions are robust to deviations from these assumptions and that generalizability of the settings is achievable.

Finally, the presented design has the potential to be applied in practice, although under certain limitations. The special characteristics of randomized test-treatment studies offer the possibility to use additional information of nuisance parameters, like the prevalence of the disease, for the recalculation of the sample size to achieve more accurate results.

For future work it would be desirable to see to what extent one can overcome the above mentioned limitations. Here, a blinded sample size recalculation based on the pooled variance in case of a continuous endpoint (or pooled proportion in case of binary endpoint) and an unblinded sample size recalculation based on the treatment effects can be a compromise that does not use as much information as the unblinded sample size adjustment, but can use information of one more nuisance parameter. In this context, it should be investigated how to deal with the type I error inflation, e.g. by using already existing methods for the multiplicity adjustment of the type I error level. However, in order to use outcome data for the adjustment of the sample size, the time period until measurement of the primary endpoint in relation to the recruitment speed is crucial. If the time until observation of the primary endpoint is quite long, there is a risk that insufficient outcome data will be available at the time of a possible interim analysis or that recruitment will be completed before sufficient data have been monitored. In our example study, the primary outcome is assessed 2 months and 6 months after randomization. In order to perform a blinded re-estimation of the sample size based on the pooled variation of the TBscore and KPS, it is necessary to ensure that all outcome data are available at an interim analysis after e.g. 50% of the intended patient number and further recruitment is stopped until then, probably leading to a delay in the study schedule. Therefore, this option would not be advisable.

## Supplementary Information

**Additional file 1: Table 1.** Scenarios of the simulation study. **Figure 1.** Results for the bias for the 1620 scenarios stratified by the difference in prevalence, the difference in sensitivity, and the difference in specificity. The relative bias for the adaptive design containing a re-estimation of the prevalence assuming *μ*_*I*+_ = 0.05 (Figure 1 (a)-(c)) and *μ*_*I*+_ = 0.1 (Figure 1 (d) – (f)) is presented. **Figure 2.** Results for the type I error for the 1620 scenarios stratified by the difference in prevalence, the difference in sensitivity, and the difference in specificity. The type I error of the fixed design and the adaptive design containing a re-estimation of the prevalence assuming *μ*_*I*+_ = 0.05 (Figure 2 (a)-(c)) and *μ*_*I*+_ = 0.1 (Figure 2 (d) – (f)) were compared to each other. The black solid lines mark 95% prediction intervals based on the Monte Carlo error in the simulations. The black dotted line mark the theoretical type I error of 5%. Whiskers are limited to the minimum and maximum value of the data. **Figure 3.** Results of the calculated sample sizes for the 1620 scenarios stratified by the difference in prevalence, the difference in sensitivity, and the difference in specificity. The initially calculated as well as adjusted sample size in the adaptive design containing a re-estimation of the prevalence and the true necessary sample size were compared to each other assuming *μ*_*I*+_ = 0.05 (Figure 3 (a)-(c)) and *μ*_*I*+_ = 1 (Figure 3 (d) – (f)). **Figure 4.** Comparison of the power of the fixed design and the adaptive design containing a blinded re-estimation of the prevalence with *Sp*_*A*_ = 0.8, *Sp*_*B*_ = 0.6, *μ*_*I*+_ = 0.05 (Figure 4(a)) and *μ*_*I*+_ = 0.1 (Figure 4(b)). The initially assumed prevalence is either over- or underestimated. **Figure 5.** Comparison of the adjusted sample size in the adaptive design containing a re-estimation of the prevalence and the initial sample size among 10,000 replications, when *Se*_*A*_ = 0.95, *Sp*_*A*_ = 0.9, *Se*_*B*_ = 0.7, *Sp*_*B*_ = 0.75 and *π* = 0.4, *π*_*assumed*_ = 0.2.

**Additional file 2.**

## Data Availability

All data used in this study are simulated and analyzed as part of the simulation study. All analyses and datasets supporting the conclusions of this article are carried out with R version 4.0.5 (2021-03-31) [33]. The R simulation code is provided in the additional files.

## Abbreviations

RCT: Randomized Controlled Trial
SSR: Sample size recalculation
KPS: Karnofsky Performance Score
