# Supplementary material for: Sample size recalculation based on the prevalence in a randomized test-treatment study
Source: BMC Med Res Methodol. 2022 Jul 25;22:205. doi: 10.1186/s12874-022-01678-7 (PMC9317230; doi:10.1186/s12874-022-01678-7)
Supplement: Supplementary file 1 — Additional file 1: Table 1. Scenarios of the simulation study. Figure 1. Results for the bias for the 1620 scenarios stratified by the difference in prevalence, the difference in sensitivity, and the difference in specificity. The relative bias for the adaptive design containing a re-estimation of the prevalence assuming μI+ = 0.05 (Figure 1 (a)-(c)) and μI+ = 0.1 (Figure 1 (d) – (f)) is presented. Figure 2. Results for the type I error for the 1620 scenarios stratified by the difference in prevalence, the difference in sensitivity, and the difference in specificity. The type I error of the fixed design and the adaptive design containing a re-estimation of the prevalence assuming μI+ = 0.05 (Figure 2 (a)-(c)) and μI+ = 0.1 (Figure 2 (d) – (f)) were compared to each other. The black solid lines mark 95% prediction intervals based on the Monte Carlo error in the simulations. The black dotted line mark the theoretical type I error of 5%. Whiskers are limited to the minimum and maximum value of the data. Figure 3. Results of the calculated sample sizes for the 1620 scenarios stratified by the difference in prevalence, the difference in sensitivity, and the difference in specificity. The initially calculated as well as adjusted sample size in the adaptive design containing a re-estimation of the prevalence and the true necessary sample size were compared to each other assuming μI+ = 0.05 (Figure 3 (a)-(c)) and μI+ = 1 (Figure 3 (d) – (f)). Figure 4. Comparison of the power of the fixed design and the adaptive design containing a blinded re-estimation of the prevalence with SpA = 0.8, SpB = 0.6, μI+ = 0.05 (Figure 4(a)) and μI+ = 0.1 (Figure 4(b)). The initially assumed prevalence is either over- or underestimated. Figure 5. Comparison of the adjusted sample size in the adaptive design containing a re-estimation of the prevalence and the initial sample size among 10,000 replications, when SeA = 0.95, SpA = 0.9, SeB = 0.7, SpB = 0.75 and π = 0.4, πassumed = 0.2. [file 12874_2022_1678_MOESM1_ESM.docx]

**Sample size recalculation based on the prevalence in a randomized test-treatment study**

**Additional file 1**

Amra Hot^1^, Norbert Benda^2^, Patrick M. Bossuyt^3^, Oke Gerke^4,5^, Werner Vach^6,7^, Antonia Zapf^1^

^*^Correspondence: a.hot@uke.de

^1^Institute of Medical Biometry and Epidemiology, University Medical Center Hamburg-Eppendorf, Christoph-Probst Weg 1, 20246 Hamburg, Germany. Full list of author information is available at the end of the article.

**Supplementary material**

Table 1: Scenarios of the simulation study

| **Parameters** | **Sample size recalculation** |
| --- | --- |
| **Size of internal pilot study** $f$ | $0.5$ |
| **True prevalence** $\pi_{true}$ | $0.3, 0.4$ |
| **Assumed prevalence** $\pi_{assumed}$ | $\pi_{true}\pm0.1,\pm0.2, +0.25$ |
| $Se_{A}$ | $0.7, 0.8, 0.9$ |
| $Sp_{A}$ | $0.7, 0.8, 0.9$ |
| **Expected outcomes** |  |
| $\mu_{I-}$ **(treatment I in non-diseased population)** | $0.2$ |
| $\mu_{I+}$(treatment I in diseased population) | $0.05, 0.1$ |
| $\mu_{II-}$ (treatment II in non-diseased population) | $0.05$ |
| $\mu_{II+}$(treatment II in diseased population) | $0.25$ |
| **Under** $\boldsymbol{H}_{\boldsymbol{0}}: \boldsymbol{\theta}_{\boldsymbol{A}}=\boldsymbol{\theta}_{\boldsymbol{B}}$ |  |
| **Theoretical significance level** $\alpha$ | $0.05$ **(two-sided)** |
| $Se_{B}$ | $Se_{A}$ |
| $Sp_{B}$ | $Sp_{A}$ |
| **Under** $\boldsymbol{H}_{\boldsymbol{1}}: \boldsymbol{\theta}_{\boldsymbol{A}}\neq\boldsymbol{\theta}_{\boldsymbol{B}}$ |  |
| **Theoretical power** $1-\beta$ | $0.8$ |
| $Se_{B}, Sp_{B}$ | 1. $Se_{A}-0.2, Sp_{A}-0.2$ 2. $Se_{A}-0.25, Sp_{A}-0.25$ 3. $Se_{A}-0.3, Sp_{A}-0.3$ |

**Adaptive Design with a re-estimation of the prevalence**


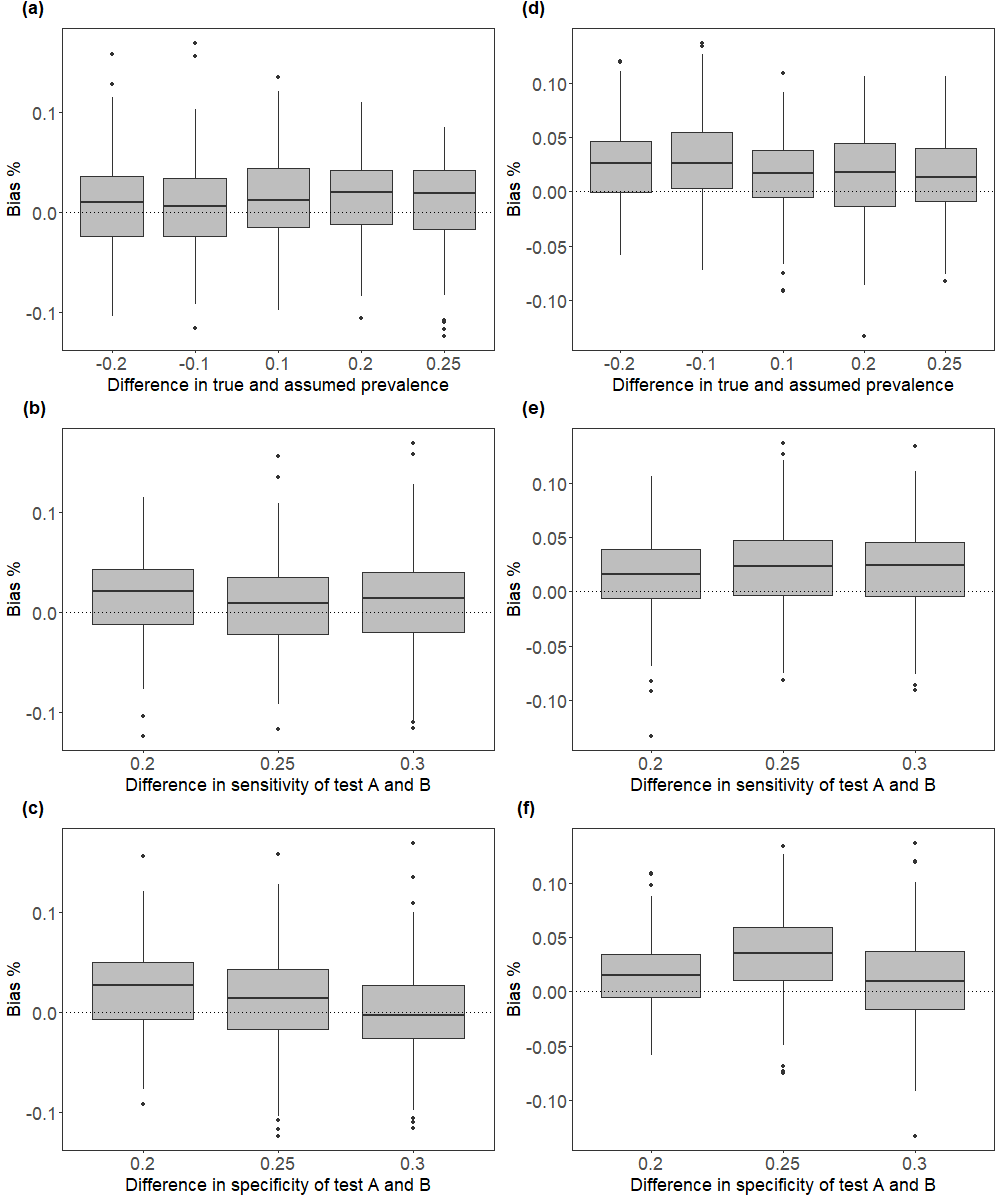


Figure 1: Results for the bias for the 1620 scenarios stratified by the difference in prevalence, the difference in sensitivity, and the difference in specificity. The relative bias for the adaptive design containing a re-estimation of the prevalence assuming $\mu_{I+}=0.05$ **(Figure 1** (a)-(c)**) and** $\mu_{I+}=0.1$ (Figure 1 (d) – (f)) is presented.


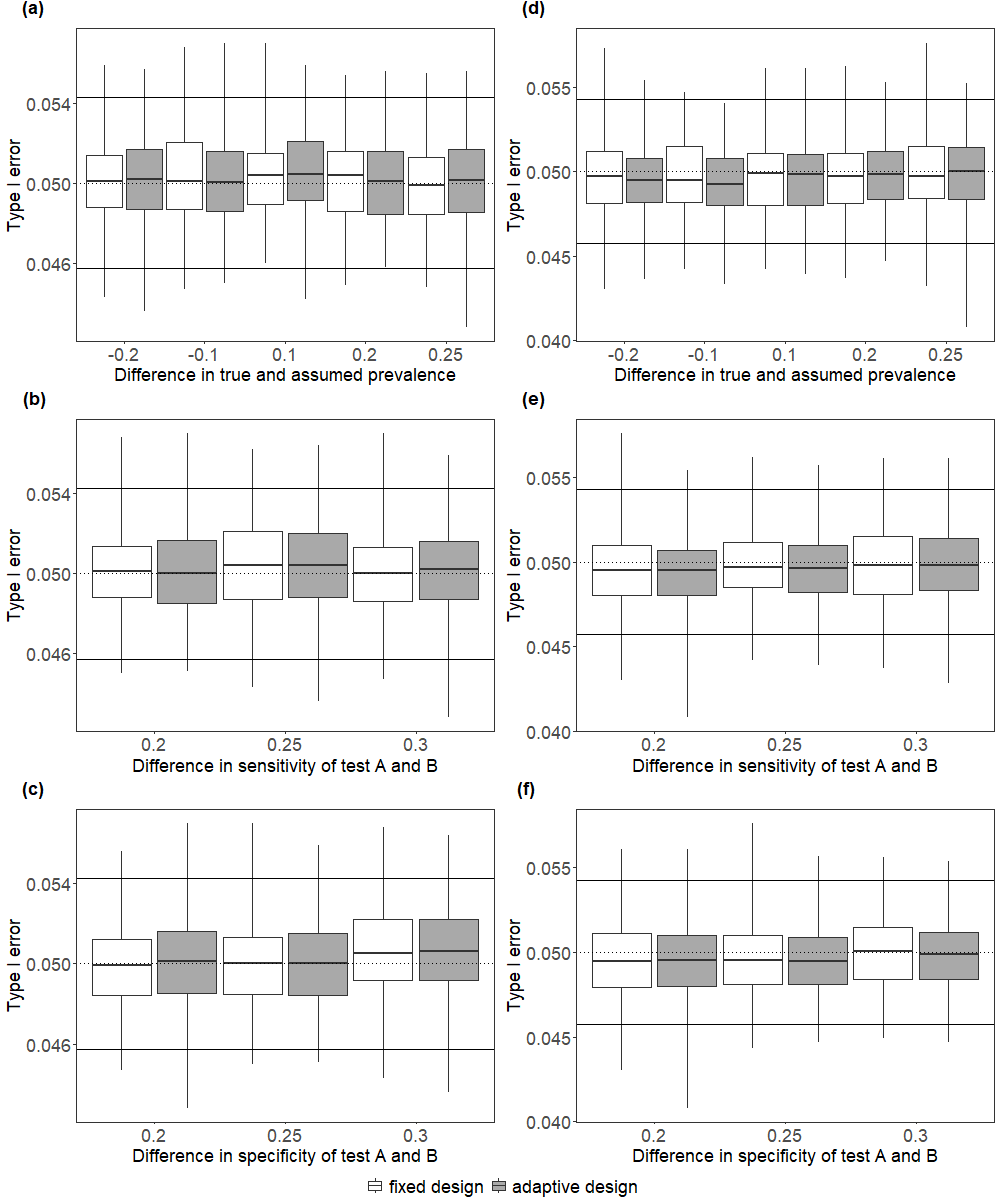


Figure 2: Results for the type I error for the 1620 scenarios stratified by the difference in prevalence, the difference in sensitivity, and the difference in specificity. The type I error of the fixed design and the adaptive design containing a re-estimation of the prevalence assuming $\mu_{I+}=0.05$ **(Figure 2** (a)-(c)**) and** $\mu_{I+}=0.1$ (Figure 2 (d) – (f)) were compared to each other. The black solid lines mark 95% prediction intervals based on the Monte Carlo error in the simulations. The black dotted line mark the theoretical type I error of 5%. Whiskers are limited to the minimum and maximum value of the data.

**
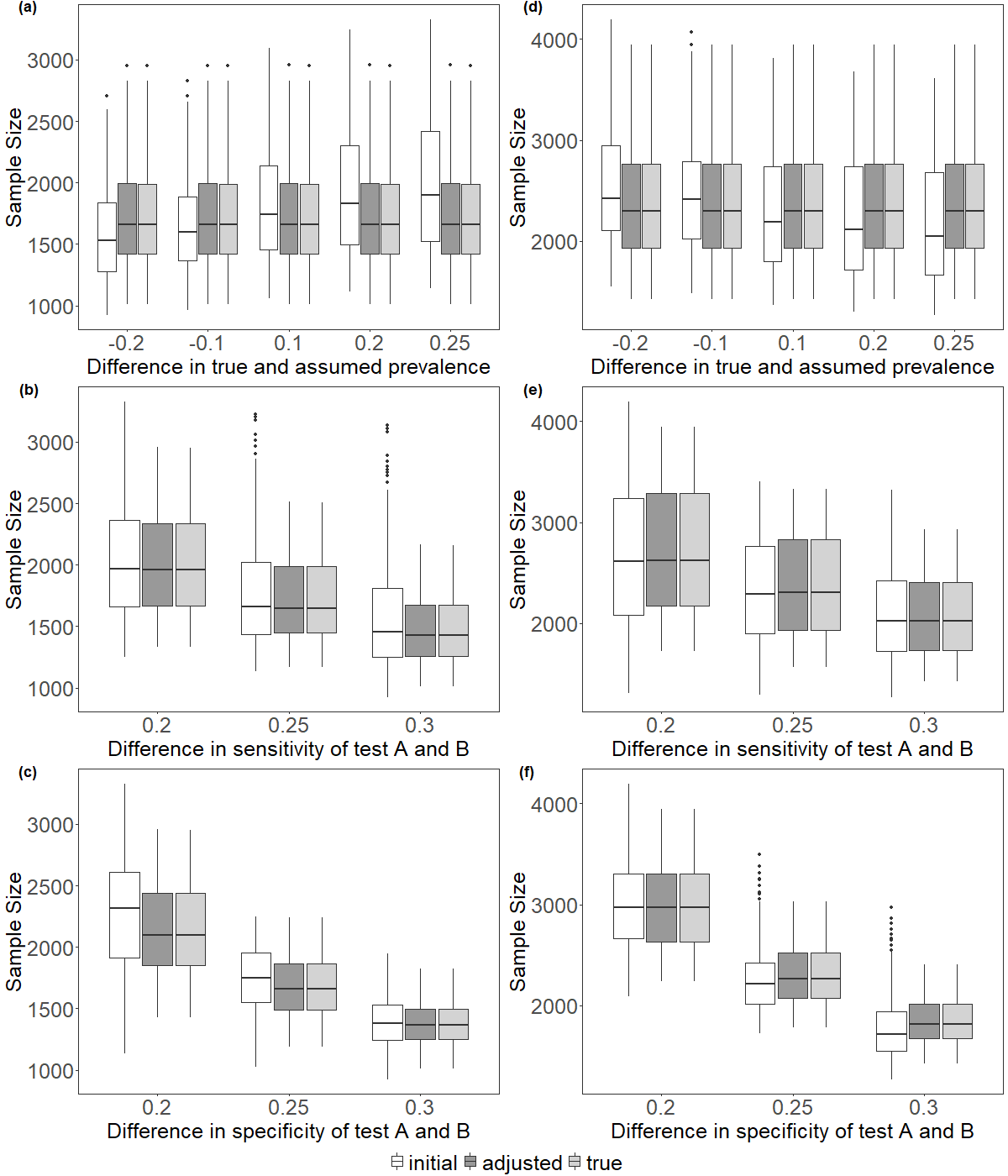
**

Figure 3: Results of the calculated sample sizes for the 1620 scenarios stratified by the difference in prevalence, the difference in sensitivity, and the difference in specificity. The initially calculated as well as adjusted sample size in the adaptive design containing a re-estimation of the prevalence and the true necessary sample size were compared to each other assuming $\mu_{I+}=0.05$ (Figure 3 (a)-(c)) and $\mu_{I+}=1$ (Figure 3 (d) – (f)).

**Adaptive Design with a re-estimation of the prevalence for selected scenarios**


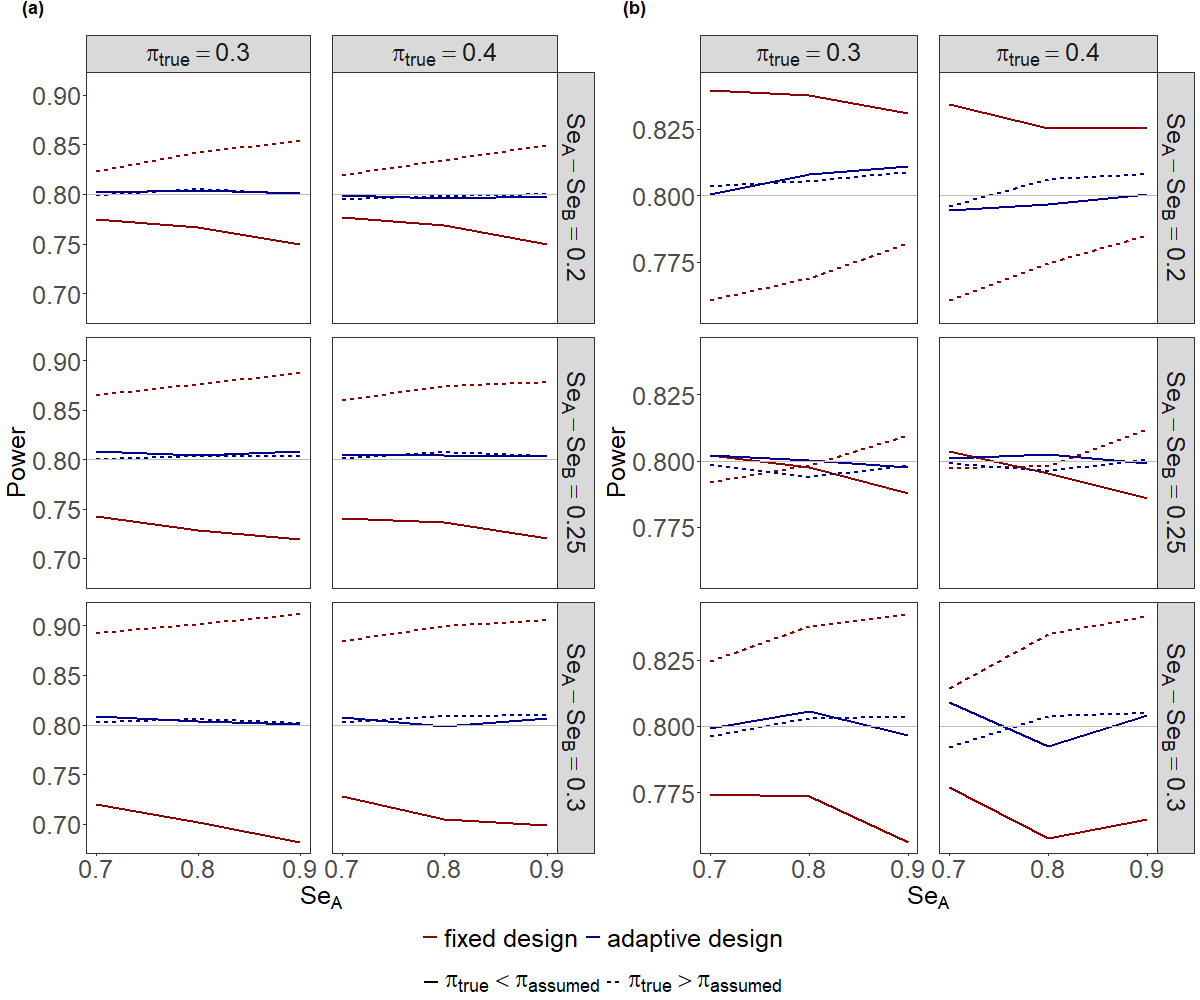


Figure 4: Comparison of the power of the fixed design and the adaptive design containing a blinded re-estimation of the prevalence with$Sp_{A}=0.8, Sp_{B}=0.6$,$\mu_{I+}=0.05$ (Figure 4(a)) and $\mu_{I+}=0.1$ (Figure 4(b)). The initially assumed prevalence is either over- or underestimated.

**
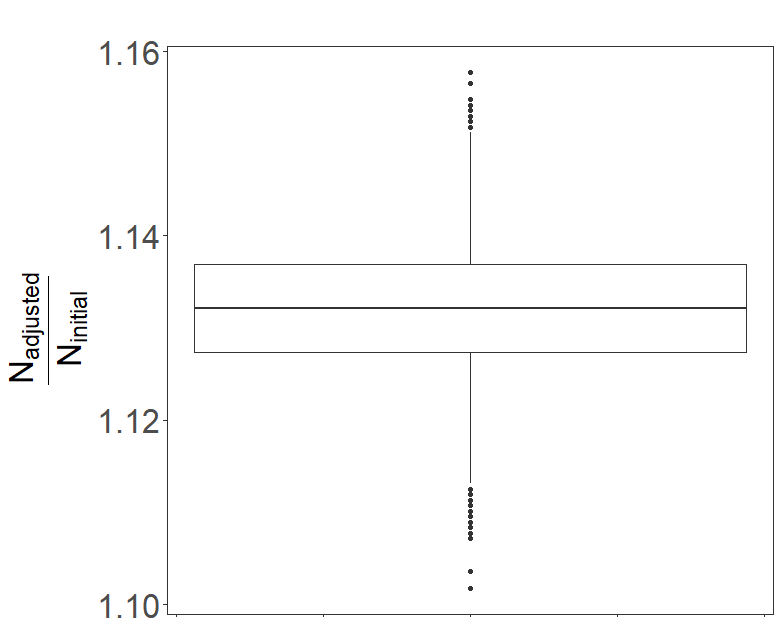
**

Figure 5: Comparison of the adjusted sample size in the adaptive design containing a re-estimation of the prevalence and the initial sample size among 10,000 replications, when $Se_{A}=0.95, Sp_{A}=0.9,$ $Se_{B}=0.7, Sp_{B}=0.75$ and $\pi=0.4, \pi_{assumed}=0.2$.

**Author’s details**

**^1^Institute of Medical Biometry and Epidemiology, University Medical Center Hamburg-Eppendorf (UKE), Christoph-Probst Weg 1, 20246 Hamburg, Germany. ^2^Federal Institute for Drugs and Medical Devices (BfArM),** **Kurt-Georg-Kiesinger-Allee 3, 53175 Bonn, Germany. ^3^Department of Epidemiology and Data Science, Amsterdam University Medical Centers, Meibergdreef 15, 1105 AZ Amsterdam, The Netherlands. ^4^Department of Nuclear Medicine, Odense University Hospital, J.B. Winsl**ø**ws Vej 4, 5000 Odense C, Denmark. ^5^Department of Clinical Research, University of Southern Denmark, Winsl**ø**wparken 19, 5000 Odense C, Denmark. ^6^Basel Academy for Quality and Research in Medicine, Steinenring 6, 4051 Basel, Switzerland. ^7^Department of Environmental Science, University of Basel, Spalenring 145, 4055 Basel, Switzerland.**
